# In Vitro and In Vivo Inhibitory Effect of Citrus Junos Tanaka Peel Extract against Oxidative Stress-Induced Apoptotic Death of Lung Cells

**DOI:** 10.3390/antiox9121231

**Published:** 2020-12-04

**Authors:** Jin Woo Kim, Eun Hee Jo, Ji Eun Moon, Hanvit Cha, Moon Han Chang, Hyung Taek Cho, Min Kook Lee, Wan Sik Jung, Jin Hyup Lee, Wan Heo, Young Jun Kim

**Affiliations:** 1 Department of Food and Biotechnology, Korea University, Sejong 8244, Korea; kju6594@korea.ac.kr (J.W.K.); cookiebb@korea.ac.kr (E.H.J.); kmseun@korea.ac.kr (J.E.M.); chahv@korea.ac.kr (H.C.); def92@korea.ac.kr (M.H.C.); jht30911@korea.ac.kr (H.T.C.); lee7641274@korea.ac.kr (M.K.L.); 2 Immunotech, Inc., Cheonan-si, Chungnam 31094, Korea; jwschj@korea.ac.kr; 3 Institutes of Natural Sciences, Korea University, Sejong 8244, Korea

**Keywords:** *Citrus junos* tanaka, oxidative stress, acrolein, apoptosis, lung

## Abstract

Various stresses derived from both internal and external oxidative environments lead to the excessive production of reactive oxygen species (ROS) causing progressive intracellular oxidative damage and ultimately cell death. The objective of this study was to evaluate the protective effects of *Citrus junos* Tanaka peel extract (CE) against oxidative-stress induced the apoptosis of lung cells and the associated mechanisms of action using in vitro and in vivo models. The protective effect of CE was evaluated in vitro in NCI-H460 human lung cells exposed to pro-oxidant H_2_O_2_. The preventive effect of CE (200 mg/kg/day, 10 days) against pulmonary injuries following acrolein inhalation (10 ppm for 12 h) was investigated using an in vivo mouse model. Herein, we demonstrated the inhibitory effect of CE against the oxidative stress-induced apoptosis of lung cells under a highly oxidative environment. The function of CE is linked with its ability to suppress ROS-dependent, p53-mediated apoptotic signaling. Furthermore, we evaluated the protective role of CE against apoptotic pulmonary injuries associated with the inhalation of acrolein, a ubiquitous and highly oxidizing environmental respiratory pollutant, through the attenuation of oxidative stress. The results indicated that CE exhibits a protective effect against the oxidative stress-induced apoptosis of lung cells in both in vitro and in vivo models.

## 1. Introduction

Reactive oxygen species (ROS) are derived from the sequential univalent reductions of molecular oxygen [1,2]. These agents are inevitably produced by internal metabolic processes associated with oxygen respiration; in particular, hydrogen peroxide (H_2_O_2_) is the most abundant ROS, which is continuously produced and accumulated intracellularly as a byproduct of the normal course of aerobic metabolism [3,4]. In addition, various environmental stresses, such as ultraviolet (UV) radiation, pathogens, and respiratory irritants, including acrolein, also lead to excessive ROS production [5,6,7]. Oxidative stress is attributed to excessive intracellular ROS formation, which ultimately inflicts apoptotic cell death via progressive oxidative damage to essential cellular components, such as nucleic acids, lipids, and proteins [8,9,10]. Hence, oxidative stress-mediated apoptotic death appears to be the main etiological factor in various pathological situations, such as neurodegenerative, cardiovascular, and lung diseases [11,12,13].

The incidence of oxidative stress-induced diseases has increased tremendously, and thus, there is a growing public interest in the supplementation of natural food products with elevated antioxidant activity and other health-promoting effects [14,15]. *Citrus junos* Tanaka, known as yuja in Korea and yuzu in Japan, is a yellow-colored citrus fruit that has been traditionally used to improve blood circulation and treat the common cold in Northeast Asia [16,17]. Recently, *C. junos* Tanaka has been found to be effective in preventing certain diseases, including those associated with oxidative stress and inflammation [17,18]. Furthermore, *C. junos* Tanaka has been also reported to reduce cancer cell growth in HT-29 colorectal cancer cells and in a mouse tumor xenograft model. Interestingly, the cancer preventive effects of *C. junos* Tanaka was closely associated with the suppression of COX-2 expression through its antioxidant activity [19]. The fruit, primarily consumed as thin slices of its peel, contains abundant vitamin C and flavonoids, including rutin, quercetin, tangeritin, and naringin [20]. Of note, the peel of *C. junos* Tanaka contains almost two-fold higher flavanone content compared to that in lemon, which ultimately exerts higher antioxidant potency [21,22]. Accordingly, we predicted that the fruit peel may provide beneficial effects, which are closely related to the improvement of respiratory health, in the oxidative stress-induced apoptotic death of lung cells owing to its potent antioxidative activity.

In the present study, we investigated whether *C. junos* Tanaka peel extract (hereinafter referred to as CE) protects lung cells against oxidative-stress induced apoptotic death as well as the potential mechanisms of action of CE when lung cells encounter an environment with high oxidative stress, such as exposure to H_2_O_2_, which is widely accepted to establish an in vitro model of oxidative stress-induced cellular damage. Furthermore, we investigated the effects and potential protective mechanisms of CE on acrolein-induced pulmonary apoptotic damages of lung tissues using an in vivo mouse model of lung disease, given the fact that the inhalation of acrolein, a ubiquitous and highly oxidizing environmental pollutant, results in the excessive generation of ROS, followed by the apoptotic death of lung cells associated with pathological pulmonary lesions [18,23]. Thus, our study extends our knowledge on the beneficial effects of CE and provides a useful model to investigate the potential application of CE for the prevention of lung disease associated with environmental toxicants, such as H_2_O_2_, in general, and acrolein, in particular.

## 2. Materials and Methods

### 2.1. Preparation of CE

*C. junos* Tanaka was provided by the Goheung County Office (Goheung, Jeolla Province, Korea), where a voucher specimen was deposited. The peels were freeze-dried, ground with a mill, and passed through a 50-mesh sieve. The ground powder was extracted in sealed baffle flask 20 times with 50% ethanol by shaking incubator (JSSI-100C, JS Research Inc., Gongju, Korea) at 200 RPM, 24 h, and at room temperature. The extract was filtered with Whatman No. 2 filter paper (Whatman Int. Ltd., Maidstone, Medway, MA, UK), freeze-dried using a programmable freeze dryer (Ilshin Lab Co., Yangju-si, Korea), and stored at −80 °C.

### 2.2. Chemical Profiling of CE

Chromatographic analytical procedures were performed on an Agilent 1260 series (Agilent Technologies, Santa Clara, CA, USA). The HPLC system was equipped with a quaternary pump, online degasser, auto plate-sampler, and photodiode array detector with an Agilent ZORBAX Eclipse XDB-C18 column (150 × 4.6 mm, 5 μm). The gradient elution system consisted of 0.1% formic acid in water (solvent A) and 0.1% formic acid in acetonitrile (solvent B), and separation was achieved using the following gradient: 10% B at 0–5 min; 10–20% B at 5–13 min; 20% B at 13–20 min; 20–50% B at 20–25 min; 50–80% B at 25–30 min. The composition was next held at 95% B for 5 min, and then returned to its initial conditions and maintained for 5 min to equilibrate the column. The detection wavelength was set at 280 nm. The column temperature was maintained at 28 °C with a thermostatically controlled column compartment. The flow rate was 1.0 mL/min and the injection volume was 10 µL.

### 2.3. Cell Culture

The biological experiments were performed using a basal epithelial cell line NCI-H460 derived from human lung carcinoma kindly provided by Prof. Song-Kyu Park (Department of Pharmacy, Korea University, Korea). The cells were cultured in Dulbecco’s modified Eagle’s medium supplemented with 10% fetal bovine serum (FBS) and 1% penicillin/streptomycin in a humidified atmosphere at 37 °C and 5% CO_2_. At approximately 70% confluence, the cells were pre-incubated with 0.5 mg/mL CE for 1 h and then incubated with 250 μM H_2_O_2_ for 24 h. The 3-(4,5-dimethylthiazol-2-yl)-2,5-diphenyltetrazolium bromide (MTT) assay was used to determine the cell cytotoxicity as previously described [24,25].

### 2.4. Animal Protocol

Experiments were performed using 8-week-old male C57BL/6J mice. The animals were housed in a temperature- and humidity-controlled cage under a 12 h light–dark cycle and had ad libitum access to a standard diet and water. All animal experiments were performed in accordance with the protocols approved by the Institutional Animal Care and Use Committee of Korea University (KUIACUC-2019-0060). The 8-week-old C57BL/6 mice, weighing approximately 21–24 g, were used for the experiments and segregated into four groups, viz., +vehicle, +CE, +vehicle/acrolein, and +CE/acrolein, with 3–6 mice per group. The mice were subjected to acute acrolein inhalation (10 ppm for 12 h) in a closed inhalation chamber in accordance with a previously published method [23,26], wherein CE (200 mg/kg) was administered to mice orally once per day for 10 days prior to acrolein exposure. After treatment with acrolein, the mice were euthanized through an inhalant anesthetic overdose of 3–5% isoflurane, and the extracted lungs were stored in a deep freezer until further experiments.

### 2.5. Hematoxylin and Eosin (H&E) Staining

For morphological assessments of lung injury, lung tissues were harvested from the mice after acrolein treatment, and then fixed with 4% paraformaldehyde and embedded in paraffin. Subsequently, 3 μm paraffin lung sections were deparaffinized, rehydrated, and stained sequentially with hematoxylin and eosin (H&E) with the standard protocol to identify air space enlargement in the lungs. The section slides were assessed by light microscopy for histological analysis.

### 2.6. DAB Staining

The 3 μm lung tissue sections were deparaffinized with xylene and an ascending ethanol series. In addition, 1 mg/mL 3,3′-diaminobenzidine (DAB, Sigma, St. Louis, MO, USA) solution (pH7.4) with 0.1M HEPES buffer and 1 mg/mL glucose was prepared for staining. Then, the slides counterstaining with hematoxylin were dehydrated in an ethanol series and mounted. Stained slides were observed by Leica light microscopy and microscope software program (Leica Application Suite, V3.3, IL, USA) for the histological image.

### 2.7. Immunofluorescence

The deparaffinized slides were incubated with anti-4-hydroxynonenal (4-HNE, Abcam) antibody at 4 °C overnight. After removing the first antibody and washing with PBS, they were incubated with Alexa Fluor 488 anti-mouse IgG antibody (ThermoFisher, Waltham, MA, USA) for 1 h at room temperature. The slides were visualized by fluorescence microscopy using the Zeiss Axiovert 200 inverted microscope. Nuclei were counterstained with 4′6-diamidino-2-phenylindole (DAPI).

### 2.8. Immunoblot Analysis

Lung tissues from mice and NCI-H460 human lung cells were homogenized with a Dounce homogenizer in lysis buffer at 4 °C. The homogenates were centrifuged at 15,000× *g* for 20 min at 4 °C, and the supernatants were transferred to different tubes, followed by measuring the protein concentrations. Samples (30 μg of protein) were subjected to 8–12% polyacrylamide gel electrophoresis. After electrophoresis, the proteins were transferred onto nitrocellulose membranes, which were incubated with primary antibodies (Cleaved poly (ADP-ribose) polymerase (PARP) (Abcam, Cambridge, MA, USA); Cleaved caspase 3, p53 upregulated modulator of apoptosis (PUMA) (Cell Signaling Technology, Danvers, MA, USA); Actin, BAX, Cytochrome c, p53 (Santa Cruz Biotechnology, Santa Cruz, CA, USA), and Prx-SO_3_ (AbFrontier, Seoul, Korea)), followed by incubation with HRP-labeled anti-rabbit IgG (ThermoScientific, Waltham, MA, USA). Immunoreactive bands were visualized using an enhanced chemiluminescence kit (Amersham Pharmacia Biotech, Amersham, Buckinghamshire, UK), and band intensities were quantified with ImageQuant 5.2 software (Molecular Dynamics Ltd., Cambridge, CB2 8PQ, UK).

### 2.9. Statistical Analysis

All experiments were repeated three times, and the data are presented as mean ± S.D. Data were analyzed by one-way ANOVA, followed by Student’s *t*-test, and *p* < 0.05 was considered statistically significant.

## 3. Results and Discussion

### 3.1. Chemical Profiling Analysis of CE

**Figure 1:**
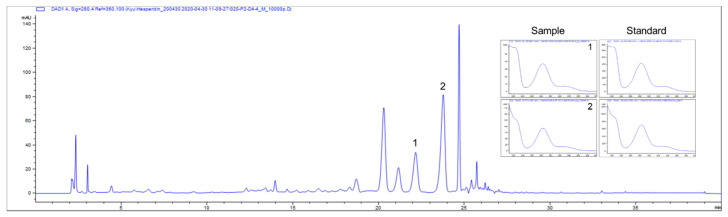
Chemical profile of *Citrus junos* Tanaka peel extract (CE). Representative HPLC chromatogram of the CE at 280 nm. The arrowed peak has been identified as naringin (1) and hesperidin (2). UV spectra of the standard and 50% ethanolic CE extract.

*C. junos* Tanaka, which is primarily consumed as thin slices of its peel, contains abundant vitamin C and flavonoids, such as hesperidin, quercetin, tangeritin, and naringin [26,27,28]. Particularly citrus flavanone glycosides, such as naringin and hesperidin, are the most abundant compounds in CE, and they are increasingly being recognized as key compounds for its role in antioxidant activities by conferring protection against oxidative stress-induced cellular damage [17,29]. Hence, the established chromatographic conditions were applied for the simultaneous determination of the main constituents in CE. With respect to the high performance liquid chromatography (HPLC) analysis, CE was standardized based on naringin and hesperidin, which is reported as a major bioactive antioxidant compound in CE [16,17,30]. As illustrated in Figure 1, naringin and hesperidin were identified as a major component of 50% ethanolic CE, as indicated by the comparison to the retention time (naringin: 22.2 min; hesperidin: 23.9 min) and absorption profile of its reference standard, and the results confirmed that CE contained 4.72 mg/g naringin and 7.53 mg/g hesperidin. [31].

### 3.2. CE Protects Lung Cells from Oxidant-Induced Cytotoxicity

**Figure 2:**
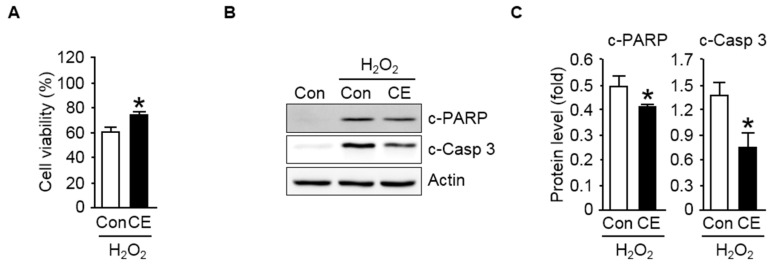
Protective effect of CE against oxidant-induced cytotoxicity in NCI-H460 human lung cells. (**A**) The viability of untreated (Con, control) NCI-H460 cells and cells treated with 0.5 mg/mL CE upon exposure to 250 μM H_2_O_2_ for 24 h was evaluated by the MTT assay. Values are represented as the mean ± S.D. from three separate experiments. * *p* < 0.05 compared with H_2_O_2_-treated cells; (**B**) immunoblot analysis of apoptosis-related proteins. The cell extracts were electrophoresed on 8–12% SDS-polyacrylamide gels, transferred to nitrocellulose membranes, and immunoblotted with antibodies against cleaved poly (ADP-ribose) polymerase (c-PARP) and cleaved caspase 3 (c-Casp 3). Actin was used as the internal control; (**C**) quantifications of the levels of c-PARP and c-Casp 3 normalized to actin are shown. Data are represented as the mean ± S.D. from three independent experiments. * *p* < 0.05 compared with the H_2_O_2_-treated group.

H_2_O_2_ is well known as a cell-damaging agent that is generated during normal cell metabolism in aerobic organisms; an excessive production of oxygen metabolites leads to oxidative stress-induced cellular damage [4,8,9]. Hence, in this study, H_2_O_2_ was used as a potentially reactive oxidizing reagent to promote intracellular oxidative stress. As apparent in Figure 2A, when the NCI-H460 human lung cell line was exposed to the pro-oxidant H_2_O_2_ (250 μM), cell viability declined drastically, resulting in higher cell mortality, suggesting the imperative role of dietary supplements in mediating a protective response of the cells against oxidative stress. Moreover, we first examined the effects of CE on the oxidative stress-induced toxicity of lung cells. To evaluate whether CE exhibits a protective effect, NCI-H460 cells were pretreated for 1 h with CE (0.5 mg/mL) prior to exposure to H_2_O_2_. As shown in Figure 2A, NCI-H460 cells with exposure to H_2_O_2_ exposited a significant decrease in viability compared to that of control cells. In contrast, the NCI-H460 cells pretreated with CE significantly exhibited the increase in the percent viability of cells compared to that of respective oxidant-treated cells, suggesting the protective role of the dietary supplements against oxidant-induced cell death.

Recently, it has been reported that lung cell cells undergo apoptotic cell death in an oxidant-induced environment, which is practically considered as the key reason behind the reduced or lost cell viability [32,33,34]. Therefore, to gain further insights into the molecular mechanisms by which CE counteracts against oxidant-induced cytotoxicity, we first examined the apoptotic markers in lung cells. As seen in Figure 2B,C, a remarkably enhanced apoptotic response was elicited in H_2_O_2_-exposed the NCI-H460 cells, as measured by the extent of the proteolytic cleavage of poly ADP ribose polymerase (PARP) and caspase 3, and importantly, the increased expression levels of apoptotic marker proteins were significantly attenuated in the NCI-H460 cells pretreated with CE.

**Figure 3:**
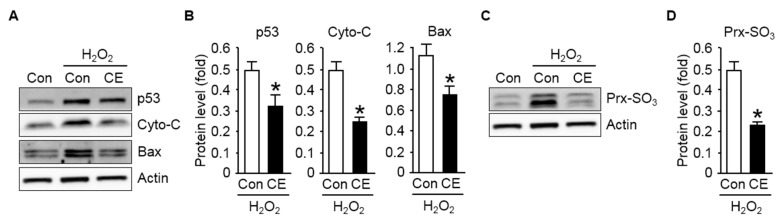
Effect of CE on p53 activation in human lung cells exposed to oxidative stress: (**A**) immunoblot analysis of p53, cytochrome c (Cyto-C), and Bax with the loading control actin; (**B**) results of p53, Cyto-C, and Bax quantification normalized to the expression of actin are shown; (**C**) immunoblot analysis of oxidized peroxiredoxin (Prx-SO_3_) levels in NCI-H460 cell lysates—actin served as the loading control; (**D**) the protein levels of Prx-SO_3_ were normalized to those of actin to quantify the immunoblotting data. All data are presented as the mean ± S.D. from three independent experiments. * *p* < 0.05 versus cells exposed to H_2_O_2_.

To further evaluate the effect of CE on the modulation of the apoptotic signaling pathway in lung cells after exposure to H_2_O_2_, we examined the activation of p53 transcription factor and the pro-apoptotic factor cytochrome c and Bax, which plays a central role in regulating apoptosis in response to various cellular stressors, such as genotoxic and oxidative stress [35,36]. Figure 3A,B indicate that the activation of p53, cytochrome c, and Bax was more pronounced when the NCI-H460 cells were exposed to H_2_O_2_. However, the increased activities of both proteins observed in the NCI-H460 cells with CE pretreatment were drastically reduced compared to that observed in control cells. This suggested that the inactivation of the apoptotic factors contributed, at least in part, to the protective effect of CE against oxidant-induced apoptotic death of lung cells.

Excessive ROS is detrimental because it causes nonspecific oxidative damage to cellular components, DNA, proteins, lipids, and other macromolecules [37,38]. In addition, elevated ROS modulates redox homeostasis and redox-regulated signaling cascades, thereby causing further oxidative stress-induced damage to tissues and cellular compartments [10]. Hence, intracellular ROS production, which exerts oxidative stress on cells, is vital for cellular stress response, including the apoptotic response. In particular, the transcription factor p53 is the primary activator that promotes apoptosis in response to intracellular oxidative stress [39]. Therefore, we examined the effect of CE on intracellular ROS production after the exposure of lung cells to H_2_O_2_ by examining the intracellular levels of oxidized peroxiredoxin (Prx-SO_3_), a well-known marker for cellular oxidative stress [40]. As depicted in Figure 3C,D, significantly increased expression levels of Prx-SO_3_ were observed in the NCI-H460 cells exposed to H_2_O_2_ compared to that in control cells, and CE pretreatment markedly reduced the occurrence of oxidative stress induced by H_2_O_2_. Together, these results indicate that the extent of apoptotic cell death in lung cells exposed to oxidative stress was significantly reduced by treating cells with CE, which is reflected by the increase in the number of viable cells under the oxidant environment.

### 3.3. CE Mitigates Acrolein-Induced Pulmonary Apoptosis in an ROS-Dependent Manner

The present study provides substantial evidence that the accumulation of ROS followed by oxidative stress-induced apoptosis was the major cause for the reduced cell viability of lung cells in an oxidant environment. More importantly, we also showed that CE supplementation is capable of decreasing intracellular ROS accumulation and p53-dependent apoptotic death while increasing the viability of lung cells exposed to the oxidant environment. These results suggested that CE supplementation could be useful in reducing pulmonary apoptotic damage associated with the oxidative stress-induced toxicity of lung tissues exposed to environmental prooxidants and toxicants, such as the inhalation of acrolein [12,23,26].

**Figure 4:**
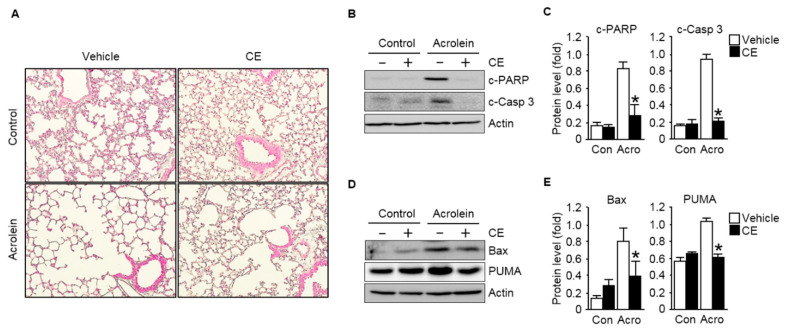
Protective effect of CE against pulmonary apoptotic injuries of lung tissues exposed to acrolein. Mice were exposed to filtered air or 10 ppm acrolein for 12 h. CE (200 mg/kg) was orally administered to mice once per day for 10 days prior to acrolein exposure. (**A**) Morphometric appearance of pulmonary tissues. H&E staining was carried out to exam the lung sections. Microscopic observations were conducted at 20× magnification; (**B**) immunoblot data comparing the levels of the apoptotic marker proteins c-PARP and c-Casp 3 in lung tissue extracts from mice. Actin was used as the loading control; (**C**) quantifications of the levels of c-PARP and c-Casp 3 normalized to those of actin are shown; (**D**) immunoblot analysis of apoptosis-related signal proteins in lung extracts exposed to acrolein with or without pre-treatment with CE. The immunoblot of actin was included as the loading control; and (**E**) the quantifications of the pro-apoptotic proteins BCL2-associated X (Bax) and p53 upregulated modulator of apoptosis (PUMA) normalized to those of actin are shown. Each value is presented as the mean ± S.D. from three to six independent experiments. * *p* < 0.05 versus acrolein-treated group. Con, control; Acro, acrolein.

Hence, as a further proof of principle that CE can be used as a therapeutic modality, the potential role of CE on acrolein-induced pulmonary apoptosis was investigated using an in vivo mouse model. The mouse model was established in 8-week-old male C57BL/6J mice orally administered CE (200 mg/kg once per day) for 10 days prior to acrolein exposure. The mice were then exposed to 10 ppm acrolein for 12 h. After the administration period, histological analyses were performed to determine the effects of acrolein inhalation on pulmonary injuries and the efficacy of CE in the treatment of the injuries. Notably, compared to the acrolein-treated group, the administration of CE markedly mitigated acrolein induced-air space enlargement, a hallmark of morphological traits for pulmonary lesion (Figure 4A). Then, to gain further insights into the mechanisms by which CE counteracts against acrolein-induced pulmonary injuries, we further examined the events of the apoptotic death by measuring the levels of apoptotic markers in the lung tissues. As shown in Figure 4B,C, the acute inhalation of acrolein increased the expression of cleaved caspase 3 and cleaved PARP, apoptotic markers, in lung tissues, whereas the CE clearly ameliorated the apoptotic signals compared to those in the only acrolein-treated group. The levels of pro-apoptotic proteins, such as Bax and PUMA, also significantly increased in the lung tissues of mice exposed to acrolein. However, the increased apoptotic activities observed drastic reduction in the tissues of acrolein-administered CE-pre-treated mice compared to those in the mice treated with acrolein alone (Figure 4D,E).

**Figure 5:**
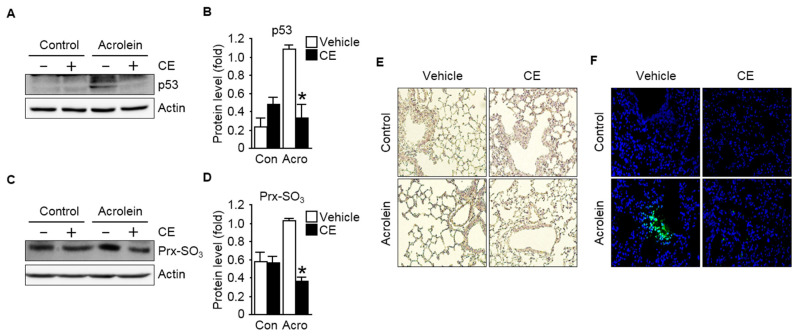
Effect of CE on p53 activation in pulmonary tissues exposed to acrolein: (**A**) p53 level in the lung sections was determined by immunoblot analysis. Actin served as an internal control; (**B**) quantification of p53 levels normalized to the expression of actin is shown; (**C**) immunoblot analysis of Prx-SO_3_ level, a marker for intracellular reactive oxygen species (ROS) formation, in acrolein-exposed lung tissues with or without pre-treatment with CE. Actin was used as the loading control; (**D**) a graph depicting the quantification of the relative abundance of the Prx-SO_3_ protein levels is shown. Each protein sample was subjected to 8–12% SDS-PAGE and immunoblotted with related antibodies as described in the text; (**E**) representative histochemical staining of the lung tissues (20× magnification) with 3,3′-diaminobenzidine (DAB) for the measurement of the level of intracellular H_2_O_2_; (**F**) representative images of immunohistochemical analyses of lipid peroxidation adducts, 4-hydroxynonenal (4-HNE) in the lung tissues (400× magnification). Nuclei were counterstained with 4′,6-diamino-2-phenylindole (DAPI). Results are expressed as the mean ± S.D. (n = 3–6 mice per group). *p* < 0.05 was considered statistically significant. * indicates statistical significance compared to the acrolein-treated group. Con, control; Acro, acrolein.

To investigate the responsible mechanisms for changes in apoptotic markers, we further assessed key markers modulating pro-apoptotic signaling pathways, such as the transcription factor p53. As presented in Figure 5A,B, the p53 protein expression level was reduced significantly by CE treatment. These results are in good agreement with the expression results of downstream proteins, such as Bax and PUMA. Previous studies have shown that acrolein induces pulmonary apoptosis via the p53 signaling pathway [26,41]. In contrast, the activation of the p53-mediated apoptotic signaling pathways is inhibited by treatment with the known antioxidant polyethylene glycol catalase [41]. This indicates that acrolein-induced intracellular ROS may be responsible for p53 signaling activation. Indeed, it is now well accepted that the apoptotic signaling pathway is susceptible to an imbalance in intracellular ROS (oxidative stress) and can be activated by it [42,43] As shown in our study, these apoptotic processes were activated by acrolein and effectively reversed by CE treatment, indicating that the apoptotic signaling pathway was blocked by CE supplementation.

As aforementioned, intracellular ROS levels are closely related with p53-mediated apoptotic signaling [35,36,39]. Considering that CE has exhibited antioxidative potential in different animal models [21,28], we examined if acrolein-induced pulmonary apoptotic damages were reversed by CE treatment through its antioxidative mechanisms associated with the activation of the p53-the dependent signaling pathway. Our observations depicted in Figure 5C,D are consistent with the hypothesis that acrolein-induced ROS generation is the main regulator to activate p53 in pulmonary apoptosis, which is in good agreement with our assessment of the higher oxidized form of peroxiredoxin in mice exposed to acrolein, as measured by the expression levels of Prx-SO_3_. In addition, intracellular reactive oxygen species and oxidative lipid damages were evaluated by measuring the levels of hydrogen peroxide (H_2_O_2_) (Figure 5E) and lipid peroxidation adduct, 4-hydroxynonenal (4-HNE) (Figure 5F), respectively. In parallel with the results in Figure 5C,D, high levels of cellular oxidative stress were observed in the lung tissues from the mice exposed to acrolein compared to those in control. More importantly, CE treatment significantly reduced the oxidative marker in tissues compared to those of mice exposed to acrolein only, suggesting that acrolein-induced oxidative damages in lung tissues were ameliorated by CE treatment in mice.

**Figure 6:**
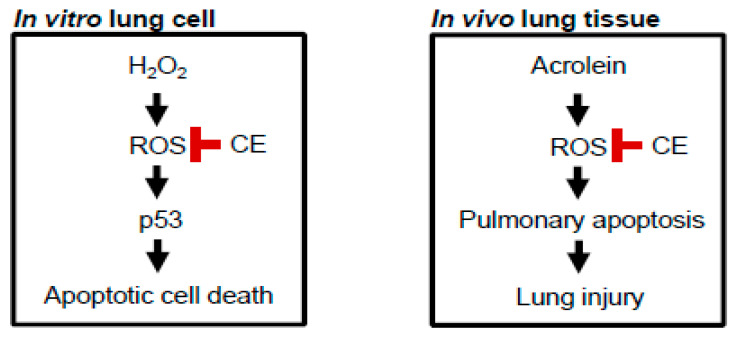
Proposed protective mechanisms of CE against oxidative stress-induced apoptotic death of lung cells in vitro and in vivo. CE antagonizes oxidative stress-induced apoptosis in lung cells exposed to the oxidant environment, thereby establishing the potential development of CE as a therapeutic agent in the treatment or prevention of pulmonary injuries associated with the exposure to highly oxidizing environmental toxicants, such as acrolein.

In summary, the present study provides evidence that the accumulation of ROS was a retarding factor for the overall survival of lung cells in an oxidant environment, such as the exposure to H_2_O_2_. More importantly, oxidative stress followed by apoptotic death was the major cause for reduced cell viability. Significantly, we also showed that CE supplementation decreased intracellular ROS accumulation and p53-dependent apoptotic signaling while increasing the viability of lung cells exposed to such an oxidant environment (Figure 6; left panel). We further investigated the protective role of CE against oxidative stress by adopting an in vivo mouse model of acrolein-induced lung damage as acrolein is known to induce oxidative stress through interactions with other intracellular molecules, owing to its high reactivity and unstable molecular structure [23,24,26]. In the present study, we were able to confirm that the apoptotic cell death observed in lung tissues upon the exposure to acrolein is closely associated with ROS formation and oxidative stress. More importantly, the antioxidative capacity of CE is primarily responsible for pulmonary protection against acrolein in mice. Furthermore, these antioxidative properties of CE successfully suppressed p53-mediated apoptotic signaling pathways, thereby alleviating acrolein-induced pulmonary lesions (Figure 6; right panel).

## 4. Conclusions

Taken together, our findings provide the evidence that CE directly antagonizes oxidative stress-induced apoptosis in lung cells, thereby establishing the potential development of CE as a therapeutic agent in the treatment or prevention of pulmonary injuries, such as pulmonary edema and chronic obstructive pulmonary disease (COPD), which are closely associated with the exposure to general and public environmental toxicants, such as acrolein [5,12,44,45]. Based on our current observations, further studies are warranted especially with regards to the protective effects of the pure compound naringin and hesperidin alone or in combination with both the phenolic compounds against the pulmonary injuries associated with environmental toxicants, such as H_2_O_2_, in general, and acrolein, in particular. Thus, the further studies could provide an insight into the current findings by which the beneficial role of CE would be clearly represented with regards to the inhibition of pulmonary apoptotic death induced by the oxidative stress.

## Data for Reference

The datasets used and/or analyzed during the current study are available from the corresponding author upon reasonable request.

## Abbreviations

CE: *Citrus junos* Tanaka peel extract; BAX: Bcl-2-associated X protein; COPD: chronic obstructive pulmonary disease; DAB: 3,3′-diaminobenzidine; FBS: fetal bovine serum; H&E: hematoxylin and eosin; H_2_O_2_: hydrogen peroxide; 4-HNE: 4-hydroxynonenal; HPLC: high performance liquid chromatography; MTT: 3-(4,5-dimethylthiazol-2-yl)-2,5-diphenyltetrazolium bromide; PARP: poly (ADP-ribose) polymerase; Prx-SO_3_: peroxiredoxin; PUMA: p53 upregulated modulator of apoptosis; ROS: reactive oxygen species; UV: ultraviolet
